# Management of Kidney Transplant Outpatients With COVID-19: A Single Center Experience

**DOI:** 10.3389/ti.2024.12920

**Published:** 2024-09-26

**Authors:** Michaela Matysková Kubišová, Sylvie Dusilová Sulková, Petr Moučka, Anita Pokorná, Marcela Heislerová, Igor Guňka, Pavel Navrátil, Jaroslav Pacovský, Alena Malá, Roman Šafránek

**Affiliations:** ^1^ Department of Nephrology, University Hospital Hradec Kralove, Hradec Králové, Czechia; ^2^ Hospital Pharmacy, University Hospital Hradec Kralove, Hradec Králové, Czechia; ^3^ Department of Surgery, University Hospital Hradec Kralove, Hradec Králové, Czechia; ^4^ Department of Urology, University Hospital Hradec Kralove, Hradec Králové, Czechia

**Keywords:** COVID-19, Sars-CoV-2, molnupiravir, kidney transplantation, antiviral drugs

## Abstract

Patients undergoing kidney transplant are at risk of severe COVID-19. Our single-center retrospective analysis evaluated the outcomes of kidney transplant outpatients with COVID-19 who were managed with reduced immunosuppression and treatment with molnupiravir. Between January 2022 and May 2023, we included 93 patients (62 men, average age 56 years), serum creatinine 127 (101–153) µmol/L. Molnupiravir was administered, and immunosuppressive therapy was reduced immediately following the confirmation of SARS-CoV-2 infection by PCR, which was 2 (1–3) days after the onset of symptoms. Only three (3.2%) patients required hospitalization, and one patient died. Acute kidney injury was observed in two patients. During the follow-up period of 19 (15–22) months, there was no significant increase in proteinuria, no acute or new chronic graft rejection, and kidney graft function remained stable; serum creatinine was 124 (106–159) µmol/L post-COVID-19 infection and 128 (101–161) µmol/L at the end of the follow-up period. Our results demonstrate that early initiation of molnupiravir treatment combined with a temporary reduction in immunosuppressive therapy results in favorable clinical outcomes in patients with COVID-19, with preservation of good graft function and no episodes of graft rejection.

## Graphical Abstract

**Figure:**
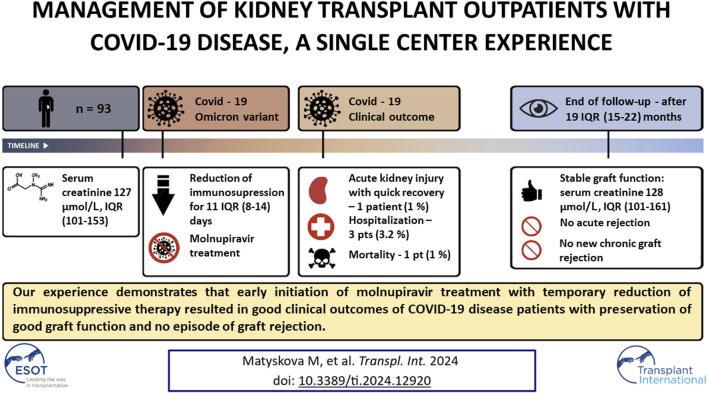

## Introduction

The SARS-CoV-2 pandemic poses a serious threat, especially for vulnerable populations, including patients who are immunocompromised, and continues to remain a major burden on the healthcare system. During the initial phase of the COVID-19 pandemic, the European Renal Association Registry reported a high mortality rate among kidney transplant recipients [1]. Subsequently, with the emergence of the Omicron variant and the introduction of various vaccinations and several antiviral drugs, the severity of the disease and hospitalization rates decreased [2]. Consequently, attention is now being paid to the management of ambulatory patients. According to the ERA Descartes Working Group, antiviral treatment is a valid option for mild-to-moderate COVID-19 in patients undergoing kidney transplant [3].

Three drugs are currently available for this purpose. However, nirmatrelvir/ritonavir was not available at the beginning of the study and has notable disadvantages, mainly due to its significant interaction with immunosuppressive drugs prescribed to patients undergoing transplantation, and the need for dosage adjustment based on kidney function. Another drug, remdesivir, must be administered intravenously and was reserved for patients with serious COVID-19. Therefore, all patients in our study were prescribed molnupiravir, which has been approved for emergency use by both the European Medicines Agency and the Food and Drug Administration. Despite its lower efficacy, molnupiravir offers significant advantages for ambulatory management; it is administered orally, lacks drug-drug interactions, and is safe for patients with a wide range of graft functions [4]. In this study, we assessed the outcomes of kidney transplant outpatients with COVID-19 managed with molnupiravir treatment in combination with temporary immunosuppressive reduction.

## Methods

SARS-CoV-2 positive kidney transplant recipients between January 2022 and May 2023 were included in this single-center retrospective study analyzing the outcomes of kidney transplant outpatients with COVID-19. The patients were followed up until February 2024. The inclusion criteria were adult, symptoms of COVID-19, and confirmed SARS-CoV-2 infection via reverse transcriptase polymerase chain reaction testing on nasopharyngeal swab specimens. All diagnoses were confirmed in the outpatient setting. Patients diagnosed with COVID-19 during hospitalization were excluded from the study. This study did not include a control group.

All patients were educated about COVID-19 symptoms and their clinical significance. They were instructed to call our transplant center immediately if they experienced COVID symptoms. Molnupiravir was administered to all patients positive for SARS-CoV-2, with none refusing treatment. Following PCR confirmation of SARS-CoV-2, molonupiravir treatment was initiated, and immunosuppressive therapy was promptly reduced. All patients were treated with a standard dose of molnupiravir capsules (800 mg every 12 h for 5 days).

Upon COVID-19 infection, immunosuppressive therapy was temporarily reduced. Mycophenolate mofetil was discontinued, and calcineurin inhibitor treatment was maintained. For patients assessed as low rejection risk by physician and with higher target tacrolimus levels (6–8 μg/L), the doses of tacrolimus was reduced to achieve target levels of 4–6 μg/L. A similar approach was used in patients treated with cyclosporine A and an mTOR inhibitor. Immunosuppressive treatment remained unchanged for patients not treated with mycophenolate mofetil, those at a higher risk of rejection, or those already receiving minimal immunosuppressive treatment. Pre-COVID-19 doses of immunosuppressive therapy were resumed after significant clinical improvement or complete resolution of symptoms.

This study aimed to assess the safety and feasibility of the described treatment approach for COVID-19 course and outcome, kidney graft function, and incidence of rejection episodes. For this purpose, we analyzed graft function prior to COVID-19 diagnosis, 2–3 weeks after the onset of the disease, and at the end of the follow-up period (February 2024). The patients were followed-up for a median duration of 19 (15–22) months. Routine monitoring during follow-up included regular assessment of excretory kidney function and quantitative proteinuria. If a clinician suspected humoral rejection, the LUMINEX method was used to detect donor-specific antibodies, and in uncertain cases, a kidney biopsy was performed. Acute kidney injury was defined according to Kidney Disease: Improving Global Outcomes guidelines as an increase in serum creatinine to 1.5 times baseline, which is known or presumed to have occurred within the prior 7 days [5]. The institutional ethics committee waived the requirement for informed consent from the patients because of the observational nature of the study.

## Results

A total of 93 patients (62 men; mean age: 56 years) were included in the study, 89% of whom had undergone their first kidney transplant. Patient characteristics are shown in Table 1. Continuous variables are presented as median and interquartile range (IQR). No patients were lost to follow-up.

**TABLE 1:** Baseline characteristics of patients.

Characteristics	Values
Patients	93
Age (years), median (IQR)	58 (46–67)
Male (years), median (IQR)	54 (45–64)
Female (years), median (IQR)	64 (50–69)
Sex	
Male, n (%)	62 (67)
Female, n (%)	31 (33)
Time from transplantation (years), median (IQR)	6 (2–11)
Previous transplant, n (%)	10 (11)
Living donor, n (%)	5 (5.4)
Baseline serum creatinine (µmol/L), median (IQR)	127 (101–153)
Diabetes, n (%)	36 (39)
Hypertension, n (%)	89 (96)
Coronary artery disease, n (%)	15 (16)
COPD, n (%)	7 (8)
BMI (kg/m^2^), median (IQR)	27.7 (25.9–31.1)
Donor specific antibodies (from 80 examined patients), n (%)	2 (3)
Vaccinated against SARS-CoV-2, n (%)	86 (93)
At least one booster dose, n (%)	61 (66)
Interval between last vaccination and COVID-19 disease (months), median (IQR)	9 (4–12)
Previous COVID-19 disease, n (%)	28 (30)
Immunosuppressive drugs in the patients	
Tacrolimus, n (%)	87 (94)
Cyclosporin A, n (%)	3 (3)
mTOR inhibitor, n (%)	3 (3)
Mycophenolate mofetil, n (%)	79 (85)
Prednisone, n (%)	92 (99)

IQR, interquartile range; COPD, chronic obstructive pulmonary disease; BMI, body mass index.

A full course of vaccination against SARS-CoV-2 was completed by 86 patients (93%). Patients were vaccinated with the available vaccines. Among them, 53 patients received the COMIRNATY, 15 patients received the Moderna COVID-19 vaccine, and 18 patients received both vaccines. Most patients received three doses of vaccination (60 patients, 65%), 25 patients (27%) received two doses, and only one patient received a single dose. The interval between the last COVID-19 vaccine dose and disease onset was 9 (4–12) months. Only three patients did not have previous COVID-19 infection nor were vaccinated. One patient developed pneumonia and recovered, whereas the other two had favorable clinical outcomes. Immunoglobulin responses to vaccination are not routinely monitored. Details regarding vaccinations are shown in Table 1.

During the entire study period, the Omicron variant was the dominant type of SARS-CoV-2 virus strain in our region. From January 2022 to January 2023, subtypes BA.1, 2, and 5 were the most common. In February 2023, subtypes BQ.x, XBB.1.x, and BN.x were prevalent. From March to May 2023, XBB.1.x was the dominant subtype.

All patients completed the prescribed course of molnupiravir treatment. There was only one reported adverse event (pruritus), which was possibly related to molnupiravir treatment; however, molnupiravir was not discontinued.

Molnupiravir treatment was initiated 2 (1–3) days after the onset of COVID-19 symptoms. A runny nose and cough were the most frequent symptoms at presentation, with only three patients experiencing shortness of breath. Detailed information regarding the symptoms is presented in Table 2.

**TABLE 2:** Common symptoms of COVID-19 at presentation.

Symptom	No. of patients (%)
Rhinitis	59 (63)
Cough	45 (48)
Fever	27 (29)
Sore throat	18 (19)
Subfebrile state	12 (13)
Fatigue	12 (13)
Muscle and joint pain	10 (11)
Headache	9 (10)

Immunosuppressive therapy was reduced in 87 (93.5%) patients. Mycophenolate was discontinued in 76 (82%) patients and reduced in 3 (3%) patients. The morning prednisone dose was increased to a median of 10 (10–15) mg, while 4 patients maintained a 5 mg dose. Immunosuppression was reduced for 11 (8–14) days. Immunosuppressive treatment was not reduced in 6 patients (6.5%).

The immunological risk in our patient population was generally low, with only three patients having donor-specific antibodies and two having known chronic humoral rejection prior to COVID-19 infection. A LUMINEX examination was performed on 46 (49%) patients during follow-up, and no new donor-specific antibodies were detected. Additionally, a kidney biopsy was indicated in three patients during follow-up and did not reveal any new cases of chronic or acute humoral rejection.

Overall, the clinical outcomes of patients with COVID-19 in our study have been good. We observed only one case of acute kidney injury, with recovery of kidney function occurring within 2 weeks. Hospitalization was required for three patients (3.2%), all due to pneumonia. The first patient, who was initially naive to COVID-19, recovered fully. The second patient had more severe symptoms and required artificial ventilation but eventually recovered. The third patient, who had acute kidney injury and graft failure, died. During the follow-up period, two patients died, and two patients required hemodialysis treatment due to causes not related to COVID-19. No episodes of acute rejection occurred during follow-up. By the end of the study, proteinuria and serum creatinine levels did not show a significant increase compared with the baseline values, as shown in Table 3.

**TABLE 3:** Parameters of transplanted kidney function.

Parameter	At baseline	After COVID-19	End of follow-up period
Creatinine (µmol/L), median (IQR)	127 (101–153)	124 (106–159)	128 (101–161)
UPCR (g/mol), median (IQR)	12 (5–27)	12 (6–28)	9.2 (4.7–24)

UPCR, urine protein/creatinine ratio. No statistically significant differences was observed between the groups (Wilcoxon signed-rank test).

## Discussion

The present study focused on the clinical outcomes of COVID-19 management using molnupiravir and a temporary reduction in immunosuppressive treatment. The clinical outcomes were positive, with only 3.2% of the patients requiring hospitalization. This rate is notably lower compared with a similar small patient group with higher hospitalization rates [6–9]. A key factor that determines the severity of COVID-19 is the type of SARS-CoV-2 virus. According to the data from our hospital laboratory, which used sequencing for viral typing, Omicron was the most prevalent variant in our region during the study period.

One of the reasons for the favorable clinical outcomes in our study might be attributed to the early initiation of antiviral treatment with molnupiravir. However, a limitation of our study was the lack of a control group to establish the benefits of this treatment. Molnupiravir has been approved for emergency use because of its demonstrated efficacy in the general population. However, studies evaluating the efficacy of molnupiravir in immunosuppressed patients are limited and have produced mixed results [10, 11]. At the beginning of our study, remdesivir was reserved for severe cases of COVID-19, and molnupiravir was the only drug available for improving the outcomes of our non-hospitalized patients undergoing transplantation. In our study, owing to the effective education of our patients and timely reporting of symptoms, we were able to diagnose COVID-19 and initiate molnupiravir treatment for most patients within 2 days of symptom onset. According to the product characteristics, treatment should be initiated within 5 days, and clinical studies suggest that earlier initiation of treatment might be related to a lower hospitalization rate [12]. Consistent with other studies, molnupiravir was well tolerated and did not show evidence of nephrotoxicity [8]. Currently, the efficacy of molnupiravir remains unconvincing, its future use is questionable, and comparative studies to other available drugs, especially nirmatrelvir/ritonavir, are lacking. Moreover, there are concerns regarding the generation of potentially transmissible molnupiravir-mutated variants [13, 14].

Vaccination against COVID-19 has been established as the most effective tool for preventing severe disease, with proven efficacy in both clinical trials and real-world settings [15]. Patients undergoing kidney transplantation were prioritized for vaccination at our center, and we achieved high vaccination rates. However, these patients were at risk of having a weak response to the vaccines. Repeated vaccinations can increase protection against severe COVID-19 [16–18]. In our study, the high rate of vaccination might have contributed to the low rate of hospitalization, with only three patients without previous COVID infection and were not vaccinated. The patient who died had accumulated risk factors, including a laboratory-proven low response to the COVID-19 vaccination. A history of previous COVID-19 infection has been recognized as a protective factor against severe disease. This trend was also observed in patients undergoing kidney transplants [19]. In our study, 28 (30%) patients had a history of prior infection. The outcome in this subgroup was favorable (no hospitalization or acute kidney injury). Our study shows that despite COVID-19 vaccination and a history of previous infection, kidney transplant recipients might not be protected from SARS-CoV-2 infection; however, their symptoms are mild, and their clinical outcomes are excellent.

The reduction of immunosuppressive medication during severe infectious diseases is routine practice in kidney transplant recipients and this approach is also applied to severe COVID-19. In the case of mild COVID-19, modification to immunosuppressive therapy may not be necessary [3]. Non-adherence and self-management issues of patients undergoing kidney transplant were also described in our previous study, highlighting the need for careful management [20]. In clinical practice, to achieve optimal results, treatment should be initiated, and immunosuppression should be reduced as soon as the diagnosis of COVID-19 is confirmed, even when the severity of the disease is uncertain. At present, there are no detailed recommendations for the modification of immunosuppressive medications during COVID-19 infection, and the practice differs among transplant centers. Commonly implemented strategies include withholding mycophenolate mofetil and slightly increasing the prednisone dose, with some studies reporting a brief period of mycophenolate mofetil withholding (e.g., 5 days) [12]. In our study, the decision to resume full immunosuppressive therapy was driven by the clinical status of the patients, with therapy being reinstated only after a significant improvement in the clinical status or complete resolution of symptoms. This resulted in a period of reduced immunosuppressive therapy for a median duration of 11 days. This approach may have enhanced the ability of the patients to fight the infection, though it also increased the risk of rejection. The reported incidence of allograft rejection after COVID-19 is highly variable among studies, though generally low [9, 21]. In line with these reports, we did not observe any acute rejection, and kidney graft function was stable during follow-up. Compared with other studies, our follow-up period was considerably longer, which allowed for the detection of changes induced by immunosuppression reduction [21, 22]. We observed a low prevalence of donor-specific antibodies (only 3%) in our study, and LUMINEX examination performed in cases of suspected humoral rejection showed no *de novo* donor-specific antibodies during follow-up.

Acute kidney injury was observed in only two patients. Although published rates of acute kidney injury are typically higher, our findings are consistent with the overall mild COVID-19 symptoms observed in our cohort [23]. The first patient with acute kidney injury had mild symptoms, and regained kidney function within 2 weeks. The second patient experienced severe symptoms and subsequently lost graft function. Similar outcomes have been reported previously. Patients with mild disease and good graft function at baseline have a high chance of graft function recovery, while patients with severe disease symptoms and poorer baseline graft function, have a higher probability of incomplete graft function recovery [24, 25].

In conclusion, we observed favorable outcomes in our group of patients. Factors contributing to these outcomes include high rates of vaccination (including booster doses), frequent prior COVID-19 infection, early initiation of antiviral treatment with molnupiravir, and early and prolonged reduction of immunosuppressive treatment. Notably, the temporary reduction of immunosuppression did not adversely affect graft function, even during long-term follow-up. Therefore, early reduction of immunosuppression appears to be an effective strategy in the management of patients with COVID-19, including those with mild symptoms at presentation.

## Data Availability

The raw data supporting the conclusions of this article will be made available by the authors, without undue reservation.
